# Physiotherapeutic Intervention in a 19-Year-Old Female Patient With Syringohydromyelia: A Case Report

**DOI:** 10.7759/cureus.50419

**Published:** 2023-12-12

**Authors:** Gunjan D Ingale, Ruchika J Zade, Rebecca P Timothy

**Affiliations:** 1 Neuro-physiotherapy, Ravi Nair Physiotherapy College, Datta Meghe Institute of Higher Education and Research, Wardha, IND

**Keywords:** atlanto-occipital c2-c3 fusion, physiotherapy rehabilitation, scoliosis, cranio-vertebral decompression, syringohydromyelia

## Abstract

Syringomyelia is a rare disorder in which a syrinx is formed in the spine that grows with time, causing damage to the entire spine. It is most commonly associated with type 1 Chiari malformations and has the potential to cause considerable impairment and a lower quality of life. It can be idiopathic or secondary to trauma. Arachnoiditis, spinal cord compression, and/or a narrow spinal canal, as well as kyphosis all play a role in the development of syringomyelia. The patient reported here was unable to walk and swallow food. She had partial sensory loss in the right hand, difficulty speaking, and weakness in both hands and legs. In this case, there was atlanto-occipital assimilation with C2-C3 fusion and tonsillar herniation, causing sryingohydromyelia of the entire cord. After medical and surgical interventions, a 12-week well-structured physical therapy rehabilitation protocol was initiated. This case study demonstrates how physical therapy plays a critical role in a patient's extensive rehabilitation, enhancing strength and range of motion, improving coordination, improving daily living tasks, and decreasing pain.

## Introduction

Syringomyelia is a spinal disorder characterised by a syrinx [1]. A syrinx is a fluid-filled cyst located inside the parenchyma of the spine or the central canal [2]. The syrinx grows and elongates with time, causing damage to the spinal cord [3]. The majority of cases are caused by an Arnold-Chiari malformation or tumours obstructing cerebrospinal fluid (CSF) circulation [4].

In some people, certain characteristics increase the risk of syringomyelia. There is an increased risk of fibrosis, which can impede CSF circulation or cause many traumatic punctures. The most significant risk factor is the presence of a full spinal cord injury [5]. Post-operative meningitis, post-subarachnoid haemorrhage (SAH), and post-myelography are all examples of post-infectious conditions. Tumours of the spinal cord, secondary myelomalacia, cord compression (herniated disc, spondylosis, tumours), infarction, and hematomyelia are a few of the causes of syringomyelia [6].

Syringomyelia-related scoliosis is a type of neuromuscular scoliosis that affects many people. Syringomyelia is a disorder marked by a diagonally aligned fluid-filled cavity in the spinal cord parenchyma that is usually related to malformation of the spine [7]. Physicians who treat patients with this disorder use a comprehensive approach to patient treatment and follow-up, forming interdisciplinary teams that include all experts who can help patients improve their quality of life [8]. Proper conservative management combined with physiotherapy helps to attain functional goals [9]. Physiotherapy is a crucial component of a patient's comprehensive rehabilitation because it improves strength and range of motion, coordination, and daily activities, as well as helps in pain relief [10].

## Case presentation

**Figure 1:**
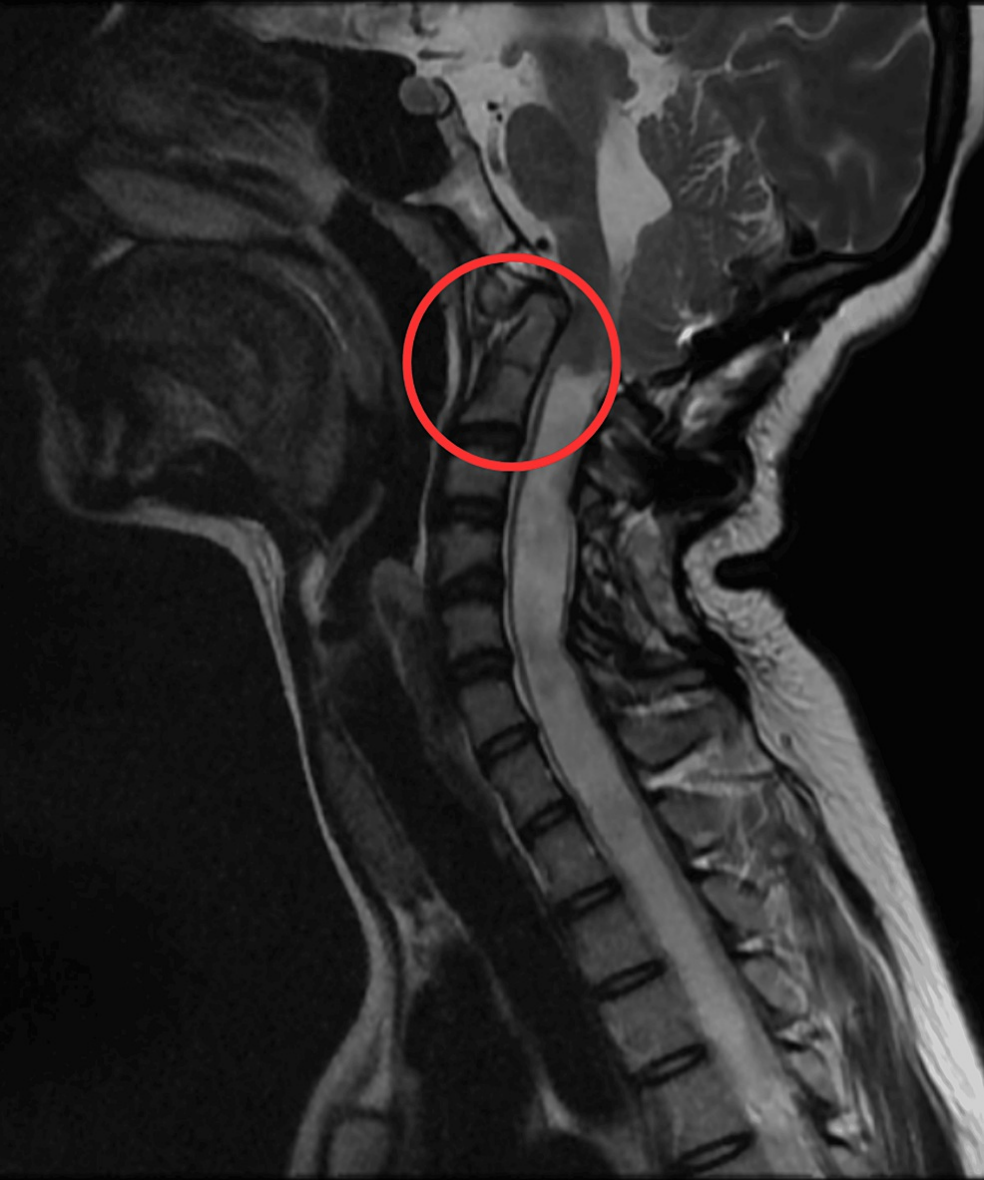
Cervical spine MRI The circle represents C2-C3 fusion and tonsillar herniation.

The patient was a 19-year-old female student with mesomorphic body type, height 1.5 m, weight 40 kg, BMI 17.77 kg/m^2^, and right-hand dominance. She complained of difficulty in walking, difficulty in swallowing, partial loss of sensation in the right hand, difficulty speaking (low speech) for two months, and weakness in both hands and legs since birth. For these complaints, she was prescribed certain medications, but since there was no relief, she was referred to the hospital for further management. A magnetic resonance imaging (MRI) scan was done that revealed atlanto-occipital assimilation with C2-C3 fusion and tonsillar herniation, causing syringohydromyelia of the entire cord (Figure 1). For the same reason, the patient underwent cranio-vertebral decompression surgery on April 10, 2022.

Clinical findings

Patient’s consent was obtained before the clinical examination began. On observation, pallor was present; no clubbing, cyanosis, oedema, icterus, or gross muscle wasting was seen in the patient. On palpation, muscle strength was reduced (Table 1). Systemic examination revealed a respiratory rate of 18 breaths per minute, with thoraco-abdominal breathing, a bilaterally symmetrical chest wall, and breath sounds normally heard. On examination, forward neck posture was noted in the lateral view, right shoulder depression was noted in the anterior view, and thoraco-lumbar spine scoliosis was noted in the posterior view. She was conscious, cooperative, and well-oriented to time, place, and person, and was answerable to all questions during higher function evaluation. Vision was intact, speech was slurred, and hoarseness of voice was present. Neurological assessment revealed tonal abnormality: Grade 1 in the right upper limb and lower limb according to the modified Ashworth Scale (MAS). All of the cranial nerves were found to be intact. Sensory examination included superficial, deep, and cortical sensations; there was partial sensory loss in the right forearm at C6, C7, and C8 levels. Reflexes were found to be normal. Romberg’s test was positive.

**Table 1:** Manual muscle testing of both upper and lower limbs pre- and post-treatment

Muscle groups	Pre-intervention		Post-intervention	
	Left	Right	Left	Right
Shoulder				
Flexors	2	2	4	4
Extensors	2	2	4	4
Abductors	3	2	5	4
Elbow				
Flexors	3	2	4	4
Extensors	2	2	4	3
Hip				
Flexors	3	2	4	4
Extensors	3	2	4	3
Abductors	2	2	4	3
Knee				
Flexors	3	2	5	4
Extensors	2	2	4	4

Non-equilibrium and equilibrium tests were performed on the patient (Tables 2, 3).

**Table 2:** Non-equilibrium tests

Non-equilibrium tests	Right limb	Left limb
Finger to finger	Good	Good
Finger to nose	Good	Good
Finger to therapist finger	Good	Good
Rebound test	Fair	Good
Pronation supination	Good	Good
Mass grasp	Good	Good
Heel to shin	Fair	Fair

**Table 3:** Equilibrium tests with eyes open and eyes close BOS: base of support

Equilibrium tests	Eyes open	Eyes close
Normal standing	Fair	Poor
Standing with narrow BOS	Poor	Poor
Standing with wide BOS	Good	Poor
Tandem standing	Poor	Poor
Standing on one leg	Poor	Poor

Therapeutic intervention

**Figure 2:**
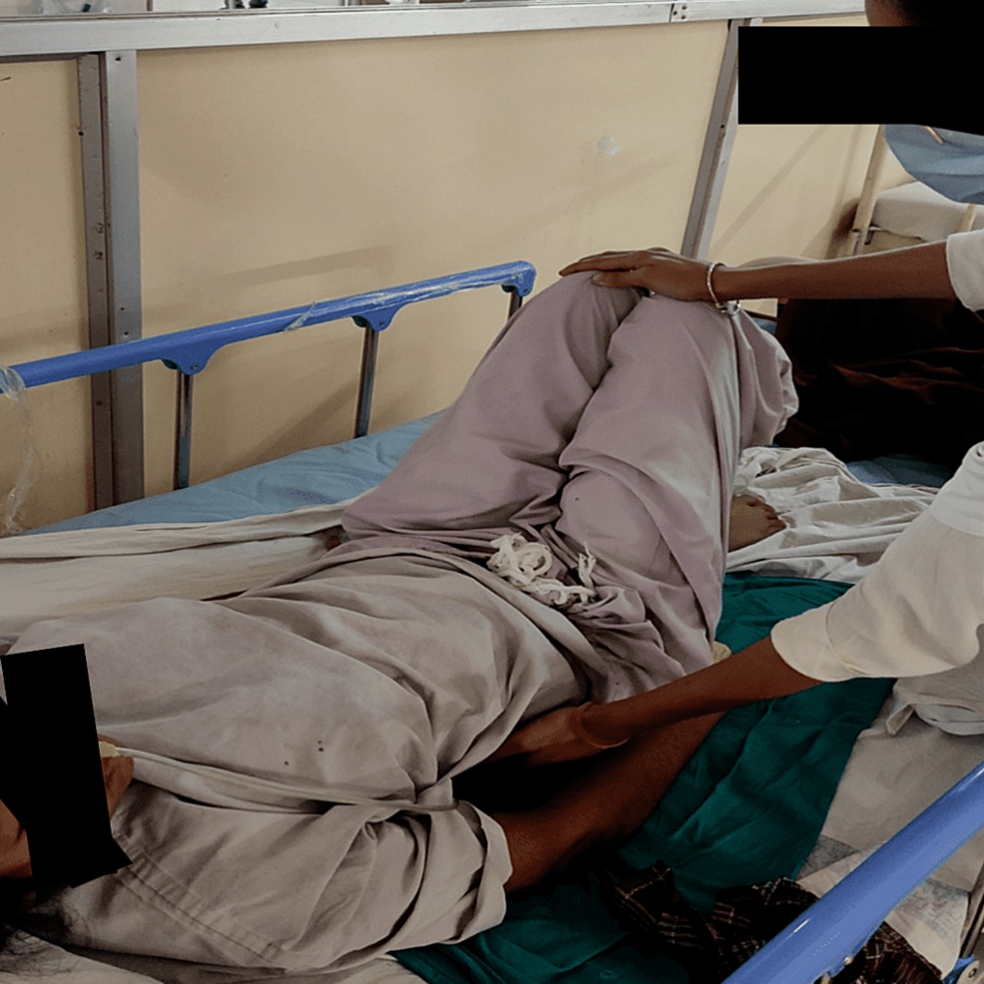
Patient performing pelvic bridging

**Figure 3:**
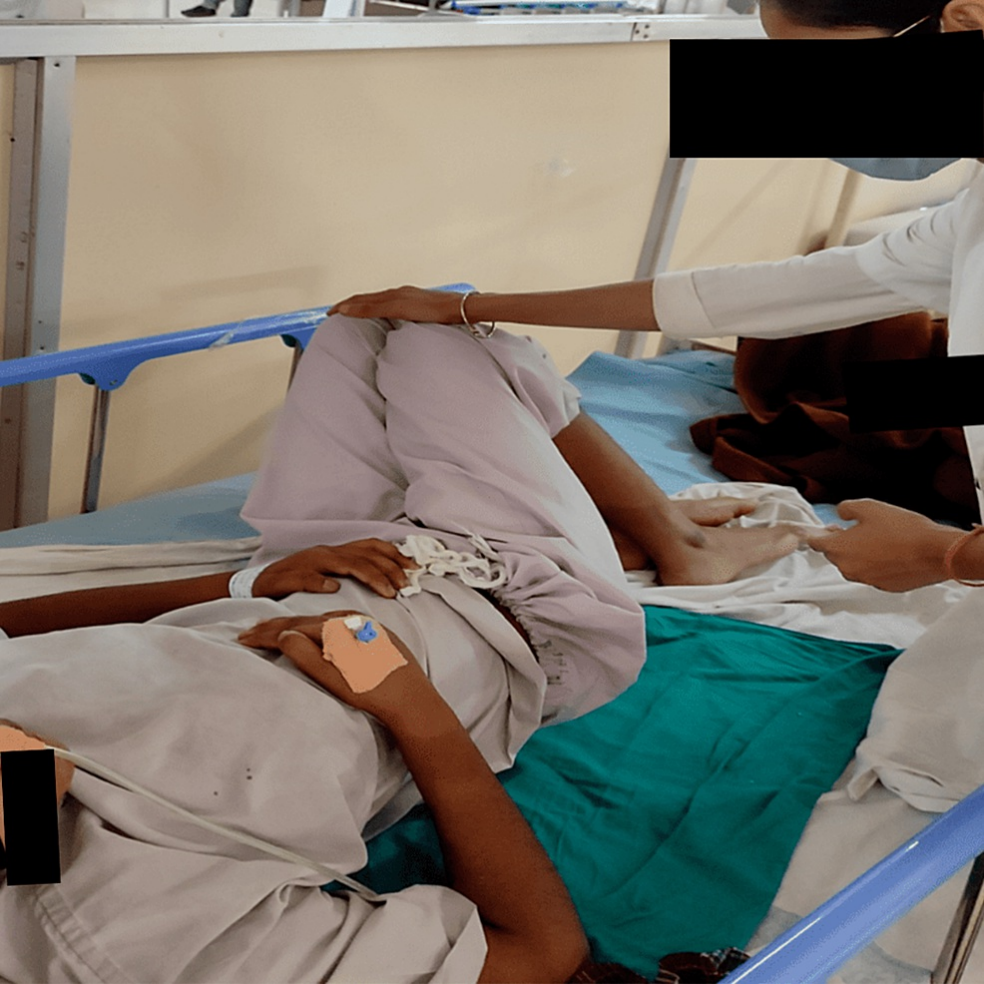
Rolling facilitation

A physical therapy rehabilitation protocol, which is described in Table 4, was planned for the patient based on the functional objectives. The primary aim was to prevent any further complications, to make the patient self-ambulatory and to improve the quality of life. The physiotherapeutic intervention is shown in Figures 2, 3.

**Table 4:** Week-wise physical therapy rehabilitation protocol ROM: range of motion, BOS: base of support, PNF: proprioceptive neuromuscular facilitation, B/L: bilateral, NS: normal saline, UL: upper limb, LL: lower limb

Intervention	Rationale	0-4 weeks	4-8 weeks	8-12 weeks
Patient education	To prevent the complication	Informing and counselling the parent about the condition of the patient and explaining the benefit of exercise		
Pain relief	To reduce pain and prevent pressure sores	To relieve pressure, pillows were placed between the legs and the positioning changed every 2 hours		
To remove the secretions	To maintain bronchial hygiene	(1) Nebulization with NS for 10 minutes with postural drainage was given to clear the secretion, (2) suctioning		
Active ROM exercises for UL and LL	To maintain and enhance the ROM	The patient was asked to perform 10 reps (three times a day) of ankle toe movement (B/L), heel slide (B/L), wrist/elbow/shoulder ROM exercises (B/L), squeezing the ball exercise	20 reps three time a day	
To improve transfers and bed mobility	To make patient independent	Mat exercise (turning to each side, rolling)		
To improve strength of upper and lower limbs	To help in transfer and ambulation	Progressive resisted exercises were given by using 0.5 kg weight with 10 reps three times a day	1 kg weight cuff with 20 reps, twice a day	1.5 kg weight cuff with 20 reps, twice a day
To improve trunk stability			Weight transfer exercises were taught, asked to sit without support for 2 min and reach out in sitting	
To improve vital capacity	To prevent pulmonary complications	(1) Chest PNF, (2) intercostal stretch	Pursed lip breathing, spirometry without hold, thoracic expansion with upper limb mobility	
To improve posture	To prevent postural abnormalities in spine	Static back with a 3-second hold, 10 reps initially; progress to 5- second hold, 20 reps	Scapular set, shoulder shrugging	Latissimus dorsi strengthening
To improve balance and coordination		(1) Finger to therapist finger, (2) heel to shin, (3) Frenkel's exercise in supine lying	Frenkel’s exercise in sitting	Sit to stand, weight shifting in standing, standing with wide to narrow BOS, reach out in standing
Ambulation				Ambulation activities in parallel bar, ambulation with assistive device

Follow-up and outcomes

Home Exercise Program

All of the exercises were asked to be continued before the patient was discharged; more emphasis was given on the respiratory system, with suggestions to perform all breathing exercises, after which we might proceed with scoliosis management. According to our findings, scoliosis was present, but we were unable to perform back-strengthening exercises owing to sutures and weakness. Table 5 shows post-intervention outcome measures.

**Table 5:** Outcome measures

Scale	1st week	4th week
Functional Independence Measure	82/126	110/126
Berg Balance Scale	32/56	46/56

## Discussion

This uncommon and understudied syndrome is characterised by progressive, restrained neurological degeneration and is thus classified as a long-term neurological condition. Physiotherapy is used in the more common disorders, and it is beneficial in improving physical, psychological, and social aspects. As a result, we hypothesise that physiotherapy may be applied to and benefit syringomyelia patients [11]. Conservative management of a patient with a cervical syrinx, using medication and physical therapy, does not reduce the size of the syrinx. Still, it can alleviate the patient's signs and symptoms by postural and biomechanical correction at the segmental level, resulting in normalisation of spinal curves and decreased tensional and compressive stress on the spinal tissue [1]. In early cases, thermography can be used to show asymmetrical sympathetic involvement [12]. The effectiveness of the McKenzie technique for the management of spinal pain was investigated in a fairly well-conducted review [13]. Even in the context of syringomyelia and Chiari malformation, spinal manipulation may be a beneficial supplementary therapeutic approach for back pain [14].

## Conclusions

This case study highlights the critical role of physical therapy in a patient's overall rehabilitation, including pain alleviation, increased strength and range of motion, improved balance and coordination, improvements in daily activities, and optimization of functional outcomes, culminating in a rehabilitation process that significantly contributes to the patient's overall recovery and well-being.

## References

[1] Osama M, Yaqoob F (2017). Cervical syringomyelia; conservative physical therapy management of a patient: a case report. Prof Med J.

[2] Roy AK, Slimack NP, Ganju A (2011). Idiopathic syringomyelia: retrospective case series, comprehensive review, and update on management. Neurosurg Focus.

[3] Mishra SS, Kimaya C, Kanchi V (2019). Effects of functional proprioceptive neuromuscular facilitation with mental practice to improve activities of daily living in syringomyelia patient - a case report. Asian J Med Sci.

[4] Lodhi MU, Kuzel AR, Syed IA, Rahim M (2017). An atypical clinical presentation of post-traumatic syringomyelia: a case report and brief review of the literature. Cureus.

[5] Giner J, Pérez López C, Hernández B, Gómez de la Riva Á, Isla A, Roda JM (2019). Update on the pathophysiology and management of syringomyelia unrelated to Chiari malformation. Neurol Engl Ed.

[6] Shenoy VS, Sampath R (2022). Syringomyelia. StatPearls [Internet].

[7] Feng F, Shen H, Chen X, Liu Z, Chen J, Li Q, Lao L (2020). Selective thoracolumbar/lumbar fusion for syringomyelia-associated scoliosis: a case-control study with Lenke 5C adolescent idiopathic scoliosis. BMC Musculoskelet Disord.

[8] Fernández AA, Guerrero AI, Martínez MI (2009). Malformations of the craniocervical junction (Chiari type I and syringomyelia: classification, diagnosis and treatment). BMC Musculoskelet Disord.

[9] Bansal R, Phanphoskar P, Wadhokar OC, Arora SP, Chitale N (2021). Physiotherapy rehabilitation in young patient with idiopathic scoliosis. J Med Pharm Allied Sci.

[10] Zade R, Sahu P, Shende G, Phansopkar P, Dadgal R (2022). Comprehensive physical therapy improves functional recovery in a rare case of stroke associated with asthma: a case report. Med Sci.

[11] Smith R, Jones G, Murphy H, Curtis A, Flint G (2015). Are established methods of physiotherapeutic management for long-term neurological conditions applicable to ‘orphan’ conditions such as syringomyelia?. Physiotherapy.

[12] Williams B (1979). Orthopaedic features in the presentation of syringomyelia. J Bone Joint Surg Br.

[13] Clare HA, Adams R, Maher CG (2004). A systematic review of efficacy of McKenzie therapy for spinal pain. Aust J Physiother.

[14] Tieppo Francio V (2014). Syringomyelia and Arnold-Chiari malformation associated with neck pain and left arm radiculopathy treated with spinal manipulation. BMJ Case Rep.

